# Using Twitter Data to Estimate the Prevalence of Symptoms of Mental Disorders in the United States During the COVID-19 Pandemic: Ecological Cohort Study

**DOI:** 10.2196/37582

**Published:** 2022-12-20

**Authors:** Ruilie Cai, Jiajia Zhang, Zhenlong Li, Chengbo Zeng, Shan Qiao, Xiaoming Li

**Affiliations:** 1 Department of Epidemiology and Biostatistics Arnold School of Public Health University of South Carolina Columbia, SC United States; 2 South Carolina SmartState Center for Healthcare Quality Arnold School of Public Health University of South Carolina Columbia, SC United States; 3 University of South Carolina Big Data Health Science Center Columbia, SC United States; 4 Geoinformation and Big Data Research Lab Department of Geography University of South Carolina Columbia, SC United States; 5 Department of Health Promotion, Education and Behavior Arnold School of Public Health University of South Carolina Columbia, SC United States

**Keywords:** mental health, anxiety disorder, depressive disorder, COVID-19, national survey, social media, Twitter, mixed model, anxiety, National Household Pulse survey, geospatial

## Abstract

**Background:**

Existing research and national surveillance data suggest an increase of the prevalence of mental disorders during the COVID-19 pandemic. Social media platforms, such as Twitter, could be a source of data for estimation owing to its real-time nature, high availability, and large geographical coverage. However, there is a dearth of studies validating the accuracy of the prevalence of mental disorders on Twitter compared to that reported by the Centers for Disease Control and Prevention (CDC).

**Objective:**

This study aims to verify the feasibility of Twitter-based prevalence of mental disorders symptoms being an instrument for prevalence estimation, where feasibility is gauged via correlations between Twitter-based prevalence of mental disorder symptoms (ie, anxiety and depressive symptoms) and that based on national surveillance data. In addition, this study aims to identify how the correlations changed over time (ie, the temporal trend).

**Methods:**

State-level prevalence of anxiety and depressive symptoms was retrieved from the national Household Pulse Survey (HPS) of the CDC from April 2020 to July 2021. Tweets were retrieved from the Twitter streaming application programming interface during the same period and were used to estimate the prevalence of symptoms of mental disorders for each state using keyword analysis. Stratified linear mixed models were used to evaluate the correlations between the Twitter-based prevalence of symptoms of mental disorders and those reported by the CDC. The magnitude and significance of model parameters were considered to evaluate the correlations. Temporal trends of correlations were tested after adding the time variable to the model. Geospatial differences were compared on the basis of random effects.

**Results:**

Pearson correlation coefficients between the overall prevalence reported by the CDC and that on Twitter for anxiety and depressive symptoms were 0.587 (*P*<.001) and 0.368 (*P*<.001), respectively. Stratified by 4 phases (ie, April 2020, August 2020, October 2020, and April 2021) defined by the HPS, linear mixed models showed that Twitter-based prevalence for anxiety symptoms had a positive and significant correlation with CDC-reported prevalence in phases 2 and 3, while a significant correlation for depressive symptoms was identified in phases 1 and 3.

**Conclusions:**

Positive correlations were identified between Twitter-based and CDC-reported prevalence, and temporal trends of these correlations were found. Geospatial differences in the prevalence of symptoms of mental disorders were found between the northern and southern United States. Findings from this study could inform future investigation on leveraging social media platforms to estimate symptoms of mental disorders and the provision of immediate prevention measures to improve health outcomes.

## Introduction

Mental disorders are a significant public health challenge in the United States. It was estimated that approximately 30% of US adults experience a disorder once in a 12-month span, and the prevalence of lifetime disorder could be as high as 50% [1,2]. Anxiety disorder is one of the most common mental disorders. The lifetime prevalence of generalized anxiety disorder ranged from 3.6% to 5.1% [2,3]. One general comorbidity of anxiety is major depression, which is another common mental disorder. The estimated prevalence of major depressive episodes was 8.12% in 2019-2020 [4]. Some previous national surveys reported that nearly 7% of adults had a symptom of depressive disorder [5-7]. Mental disorders increase the burden of socioeconomics and health care usage [8-10]. Depressive disorders and anxiety disorders were reported to be the 13th and 24th leading contributors to the global burden in 2019, respectively [11].

The COVID-19 pandemic posed significant challenges to people’s psychosocial well-being and mental health. On the one hand, the rapid transmission and variation of SARS-CoV-2 resulted in a high disease incidence and considerable mortality [12,13], which may increase fear toward COVID-19. On the other hand, myths and misinformation on social media, mandatory government responses such as citywide lockdowns, and discrimination and stigma against people infected with COVID-19 would exacerbate the mental health problems in the public [14,15]. Therefore, during the COVID-19 pandemic, anxiety and depressive disorders were common health problems among not only patients with COVID-19 [16,17] and those with pre-existing psychiatry disorders [18,19] but also the general population [20,21]. Furthermore, as a result of the increased workload in the health care system caused by the COVID-19 pandemic, an increasing number of health care providers are experiencing mental disorders [22-24]. Thus, during the outbreak period, monitoring and estimating the prevalence of mental disorders is critical to inform immediate prevention measures, reduce health care costs, and improve population health.

Social media platforms, such as Twitter, can be a potentially useful tool for estimating the prevalence of mental disorders. Messages posted on social media platforms (eg, tweets) expressing negative emotions may provide researchers with hints about the prevalence of mental disorders. Given the time-intensiveness of and high costs in recruiting participants and conducting surveys or screening using existing scales, a social media–based approach can provide timely estimation and prediction at a lower cost. This timeliness is especially a desperate need during the COVID-19 pandemic for immediate public health practices.

Social media data can provide population-wide information for estimating the prevalence of mental disorders. Previous research has taken advantage of the large volume of social media data for mental health research on public sentiment [25], estimating suicide risk in a particular population [26], or identifying risk factors and linguistic features [27,28]. Although social media data have been used for the estimation of various health issues [6,28,29], there is a dearth of research investigating whether social media can be a good data source for estimating the mental health status of the public during the COVID-19 pandemic. If it is feasible to use social media as a good instrument for estimating symptoms of mental disorders, time and resources could be saved, and more action could be taken for intervention.

To address this knowledge gap, we conducted an ecological cohort study that aims to (1) verify the feasibility of Twitter-based prevalence of the symptoms of mental disorders being an instrument for prevalence estimation, where the feasibility is gauged via the correlations between Twitter-based prevalence of symptoms (ie, anxiety and depressive symptoms) and those reported by the national surveillance data; and (2) examine the temporal trends of these correlations during different phases of the COVID-19 pandemic. Twitter is used because of its “open data” policy so that its database can be easily accessed by the official Twitter streaming application programming interface [30].

## Methods

### Data Resources and Preprocessing

The national prevalence of the symptoms of mental disorders was collected through the Household Pulse Survey (HPS), which was conducted by the National Center for Health Statistics and the Census Bureau [31]. Briefly, the HPS is a 20-minute web-based survey using a probability-based sample design to measure the social and economic impact of the COVID-19 pandemic, including the anxiety and depression status of individuals in US households; samples were chosen from the Census Bureau Master Address File Data, housing units were randomly selected to participate, and one respondent from each housing unit was selected to respond for him- or herself [32]. Measures of depressive and anxiety symptoms were based on the validated 2-item Patient Health Questionnaire and 2-item Generalized Anxiety Disorder scale, respectively. Adults were confirmed as having disorder symptoms if items included in the questionnaire generally occurred on more than half of the days or nearly every day during the past 7 days [33]. In this study, the data were aggregated by state, and collection periods consisted of 4 phases that occurred weekly from April 23 to July 21, 2020 (phase 1), biweekly from August 19 to October 26, 2020 (phase 2), biweekly from October 28 to December 21, 2020 (phase 3), biweekly from January 6 to March 29, 2021 (phase 3), and biweekly from April 14 to July 5, 2021 (phase 3.1), and the data were recorded at the end of a week.

Social media data were collected from the Twitter platform using the official Twitter streaming application programming interface from April 23, 2020, to July 5, 2021. The data were collected in JSON format. The following information was extracted: tweet ID, user ID, posted date, message, and location (to determine which state a tweet originated from). In total, approximately 5 million (N=5,099,770) Twitter users were sampled. Nonhuman user accounts (bots) were excluded following the procedure detailed in a previous study [34]. According to previous research [29,35], keywords related to anxiety and depressive symptoms were used to label the tweets and estimate the weekly or biweekly prevalence of symptoms of anxiety, depression, and overall mental disorders (anxiety or depressive symptoms) for each state at each phase provided in the HPS. The total number of users after keyword filtering was 538,801. Table 1 shows the keywords used for labeling. The prevalence of symptoms during the 4 phases was calculated as the proportion of Twitter users who had keyword-filtered anxiety and depressive symptoms tweets.

**Table 1:** List of keywords^a^.

Keywords related to anxiety symptoms	Keywords related to depressive symptoms
Anxiety	depression
Anxious	depressed
Irritable	depressive
Restless	feeling blue
Feared	insomnia
Scared	clonazepam
Nervous	imipramine
Outrage	prozac
Dread	sertraline
panic	zoloft
	hopeless

^a^The keyword list is based on Jashinsky et al [29] and Homan et al [35].

### Statistical Analysis

After data preprocessing, each state had 2 sets of data (ie, both CDC-reported prevalence and Twitter-based prevalence) for symptoms of anxiety, depression, and overall mental disorders (anxiety or depressive symptoms) at each time point. The associations between CDC-reported and Twitter-based prevalence were examined using Pearson correlation analysis. The temporal trends between them were further examined through linear mixed models with random intercepts. In linear mixed models, we used CDC-reported prevalence in each state as the outcome and Twitter-based prevalence in each state and time (in weeks) as covariates. The different measures in each state were accounted for by the normal random intercept at the state level. The interaction between Twitter-based prevalence and time was further investigated and determined with the best-subset selection based on the Akaike information criterion. Restricted maximum likelihood was used for parameter estimation. Geospatial differences were compared on the basis of random effects. Analysis was conducted in R (version 4.0.3; R Foundation for Statistical Computing) and GeoPandas. The significant level was set as *α*=.05 (2-sided).

## Results

### Descriptive Statistics

Overall, 49 contiguous states, except for Alaska and Hawaii, were included for the analysis. Table 2 summarizes the CDC-reported and Twitter-based prevalence of symptoms of anxiety, depression, and overall mental disorders across the 4 phases. According to the CDC report, nearly 30% of the participants in different states experienced symptoms of anxiety, while 24% of participants experienced symptoms of depression, and 35% of participants experienced overall symptoms of mental disorders. On Twitter, 1% of Twitter users discussed the topic of anxiety, while 0.27% of them discussed depression, and 1.3% of them discussed overall mental disorders.

**Table 2:** Descriptive statistics for the symptoms of mental disorders.

	Centers for Disease Control and Prevention			Twitter		
	Anxiety (%), median (IQR)	Depression (%), median (IQR)	Overall^a^ (%), median (IQR)	Anxiety (%), median (IQR)	Depression (%), median (IQR)	Overall^a^ (%), median (IQR)
Phase 1^b^	30.60 (27.70-33.70)	24.75 (22.10-27.60)	35.05 (32.60-38.60)	1.21 (1.10-1.34)	0.29 (0.25-0.33)	1.47 (1.34-1.61)
Phase 2^b^	31.50 (29.20-33.70)	23.90 (21.80-26.10)	36.10 (33.30-38.40)	1.20 (1.11-1.30)	0.30 (0.26-0.34)	1.47 (1.35-1.59)
Phase 3^b^	34.40 (31.60-37.10)	27.30 (24.70-29.80)	39.60 (36.60-42.30)	1.03 (0.93-1.16)	0.27 (0.23-0.31)	1.26 (1.15-1.41)
Phase 3.1^b^	25.20 (23.00-27.50)	20.90 (18.70-22.90)	29.75 (29.00-32.20)	0.92 (0.81-1.02)	0.22 (0.19-0.24)	1.11 (1.01-1.22)

^a^Anxiety or depressive symptoms.

^b^Phase 1: April 23 to July 21, 2020; phase 2: August 19 to October 26, 2020; phase 3: October 28 to December 21, 2020, and January 6 to March 29, 2021; and phase 3.1: April 14 to July 5, 2021.

### Correlation Analyses

**Figure 1:**
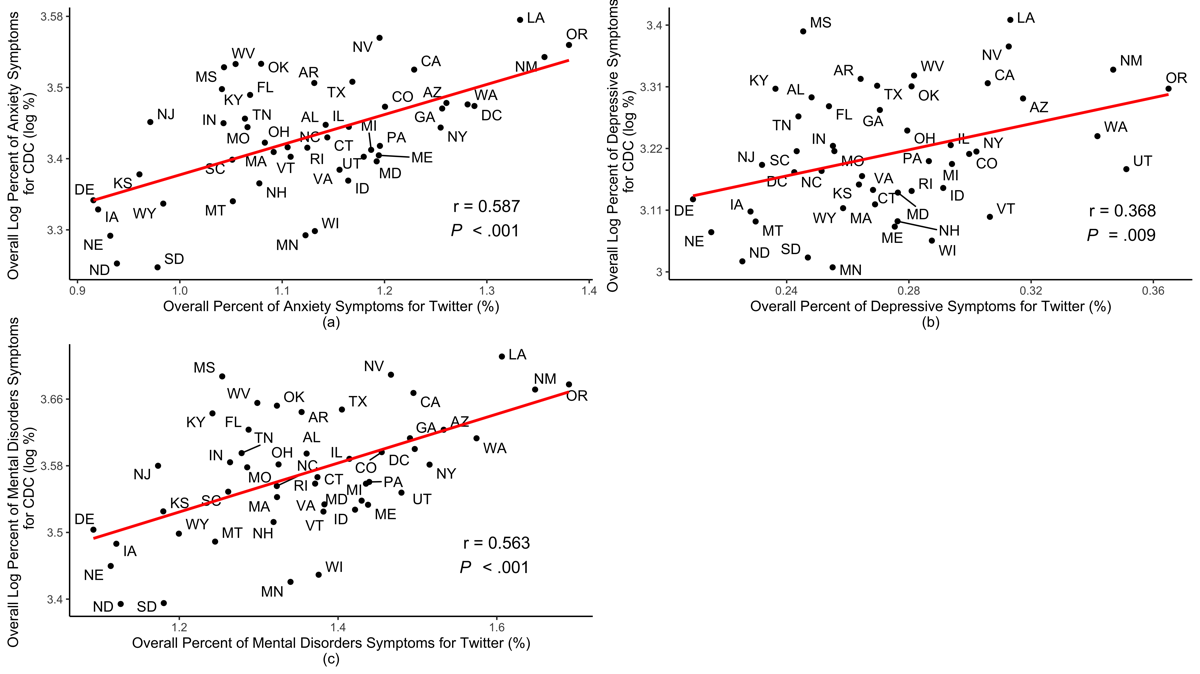
Correlations between Twitter-based prevalence and the CDC-reported prevalence of symptoms of (A) anxiety, (B) depression, and (C) overall mental disorders. CDC: Centers for Disease Control and Prevention.

Among the 49 states, the overall (anxiety or depressive symptoms) Twitter-based prevalence of symptoms of mental disorders showed a significant positive correlation with CDC-reported prevalence. Specifically, as shown in Figure 1, Pearson correlation coefficients for the prevalence of symptoms of anxiety, depression, and mental disorders reported by the CDC and estimated using Twitter were 0.587 (*P*<.001), 0.368 (*P*<.001), and 0.563 (*P*<.001), respectively.

### Regression Analysis

Models with predictors including time, Twitter-based prevalence, and the interaction between them had the lowest Akaike information criterion for symptoms of anxiety, depression, and overall mental disorders, which was used as the final model. Detailed coefficients and numbers of repeat-measured observations of each model are reported in Multimedia Appendix 1.

**Figure 2:**
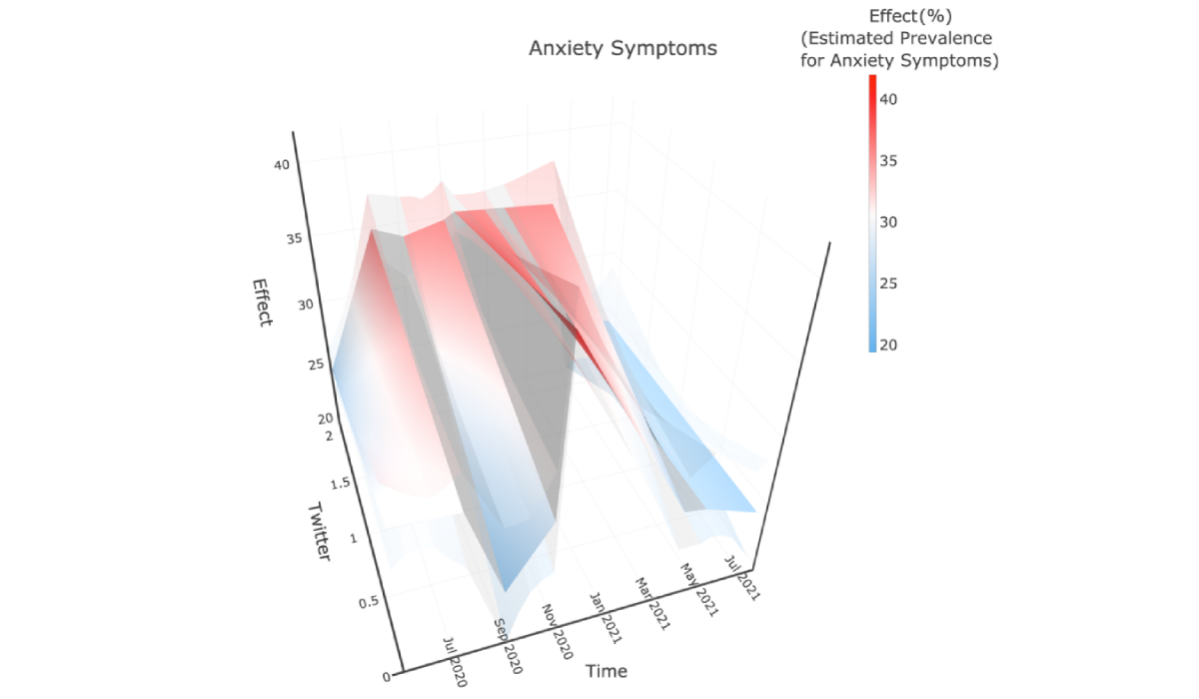
Model-based mean function of CDC-reported prevalence of anxiety symptoms. The “Time” axis shows the study period; the “Twitter” axis shows the range of Twitter-based prevalence for anxiety symptoms; and the “Effect” axis represents the mean value of model-estimated prevalence of anxiety symptoms. The solid surface in the middle was the mean value and translucent surfaces above and below showed the upper and lower bounds of the 95% CI.

**Figure 3:**
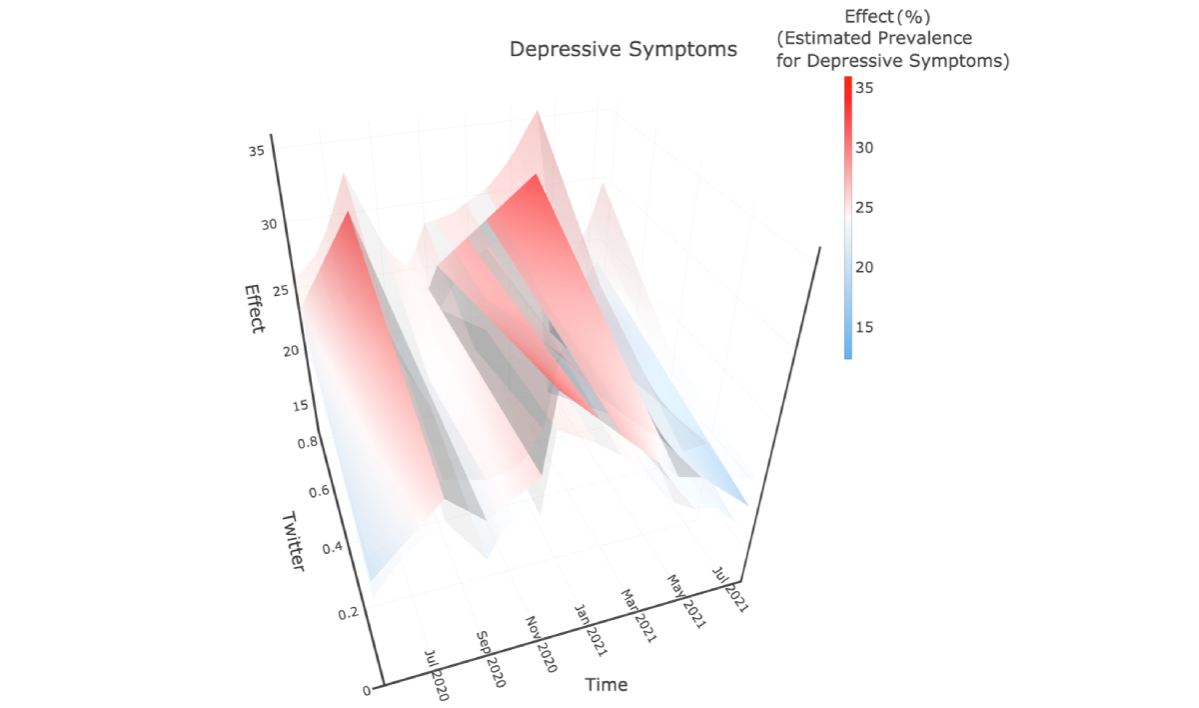
Model-based mean function of CDC-reported prevalence of depressive symptoms. The “Time” axis shows the study period; the “Twitter” axis shows the range of Twitter-based prevalence for depressive symptoms; and the “Effect” axis represents the mean value of model-estimated prevalence of depressive symptoms. The solid surface in the middle was the mean value and translucent surfaces above and below showed the upper and lower bounds of the 95% CI.

**Figure 4:**
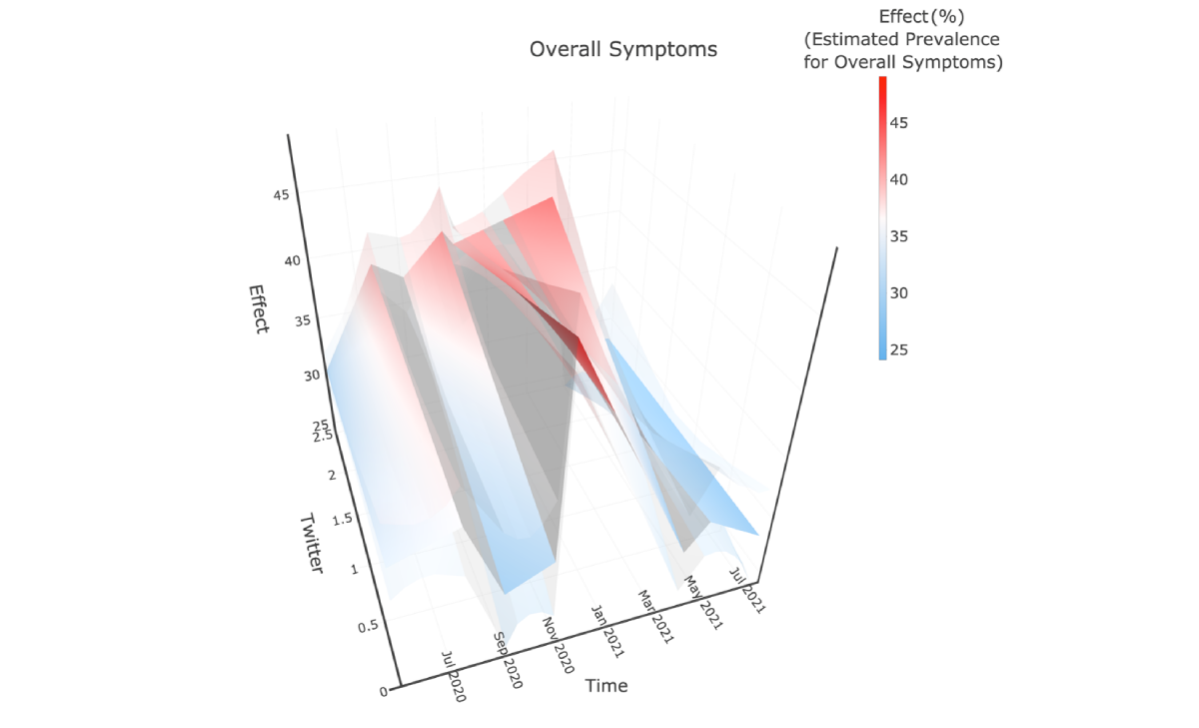
Model-based mean function of CDC-reported prevalence of symptoms of overall mental disorders. The “Time” axis shows the study period; the “Twitter” axis shows the range of Twitter-based prevalence of symptoms of mental disorders; and the “Effect” axis represents the mean value of model-estimated prevalence of symptoms. The solid surface in the middle was the mean value and translucent surfaces above and below showed the upper and lower bounds of the 95% CI.

The 3-D plots (Figures 2-4) were used to illustrate the interplay among the fitted CDC-reported prevalence, Twitter-based prevalence, and time. In Figures 2-4, the x-axis, “Time,” indicates that the study period that was from May 5, 2020, to July 5, 2021, and the y-axis, “Twitter,” showed the range of Twitter-based prevalence for anxiety symptoms (as %); the y-axis, “Effect,” represents the mean value of model-estimated prevalence of anxiety symptoms (in %). The solid surface in the middle was the mean value, and translucent surfaces above and below show the upper and lower bounds of the 95% CI.

### Anxiety Symptoms

In general, anxiety symptoms increased until November 2020 and then decreased rapidly. The largest fitted prevalence was found at the beginning of phase 3, which is shown as the reddest surface. To clarify the relationship, the 2D association of Twitter-based prevalence and estimated CDC-reported prevalence is drawn in Multimedia Appendix 2. We obtained negative but insignificant coefficients at the beginning of phases 1 and 3, while significant positive coefficients were obtained at phase 2 and the rest of phases 1 and 3.

### Depressive Symptoms

The model-estimated prevalence was initially low, increased rapidly in phase 1, but decreased slightly in phase 2. In phase 3, the prevalence was maintained at a high level but plummeted at the beginning of phase 3.1. The pattern is further shown in Multimedia Appendix 3, which indicates that Twitter-based prevalence had a significant and positive correlation with CDC-reported prevalence only in phase 1 and the second part of phase 3. Correspondingly, in Figure 3, temporal trends in correlations are shown through color variation from white to red in phase 1 and in the second part of phase 3.

### Symptoms of Overall Mental Disorders

The surfaces of fitted CDC-reported prevalence of the symptoms of overall mental disorders were similar to those for anxiety symptoms. The marginal coefficient of the Twitter-based prevalence had a decreasing trend for anxiety symptoms but had an increasing trend for symptoms of overall mental disorders in phase 2, while both of them showed positive correlations (Multimedia Appendix 4).

### Spatial Patterns of the Random Effects

**Figure 5:**
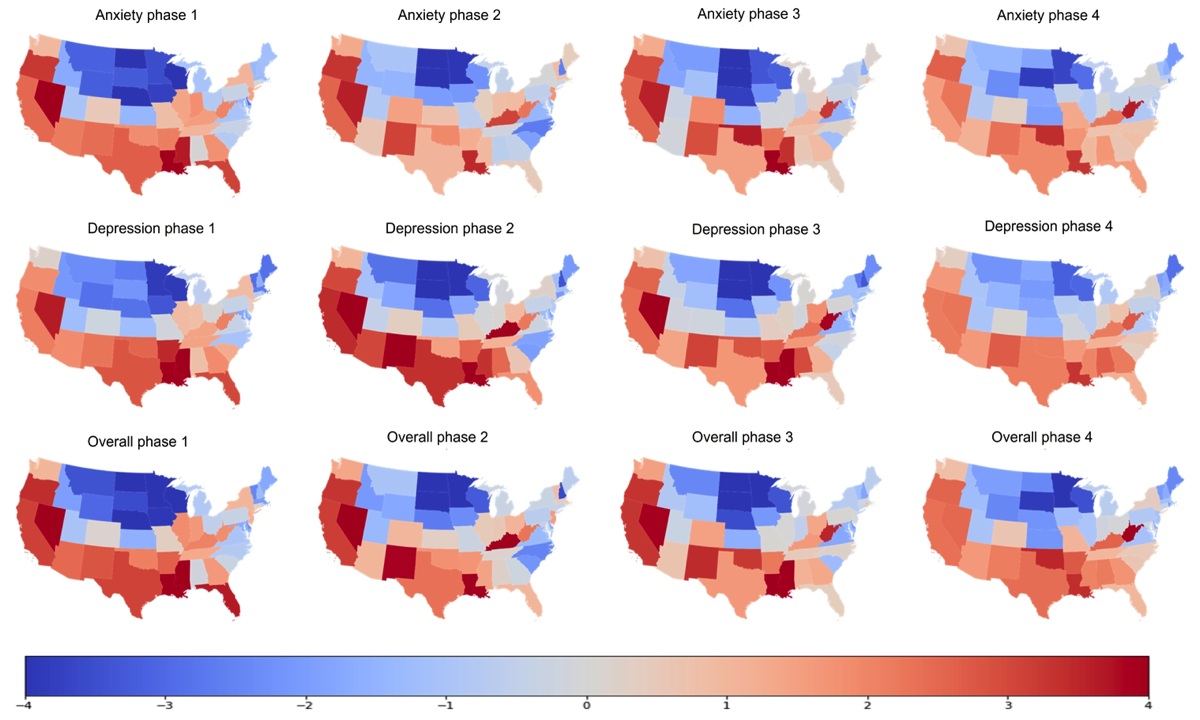
Spatial patterns of the random effects for different phases.

Geospatial disparities in the prevalence of symptoms of mental disorders across the states were characterized by the random effects of linear mixed models. A larger random effect indicated a larger deviance of a state from the nationwide average. To show the geospatial disparities, maps with random effects were drawn (Figure 5). A clear geospatial difference in the prevalence of symptoms of mental disorders between the northern and southern United States was found. Specifically, states located in the north-central and northeast United States had relatively lower mental health prevalence than those in the southern and southwestern United States across the 4 phases and 2 types of symptoms of mental disorders. As shown in the fourth column in Figure 5, the colors are lighter and the random effects are closer to 0, which indicates that the disparity decreased in phase 3.1.

## Discussion

### Principal Findings

This study examined the correlations in the prevalence of symptoms of mental disorders between those reported by the CDC and those estimated using Twitter data, as well as the temporal trends of these correlations. We note that the results of our study only capture the relationship among symptoms of mental disorders and do not imply mental disorders, since the CDC-reported prevalence was based on the 2-item Patient Health Questionnaire and the 2-item Generalized Anxiety Disorder scale. These findings indicate medium positive correlations between CDC-reported and Twitter-based prevalence for anxiety and overall mental disorder symptoms, and a weaker positive correlation for depressive symptoms. These correlations varied by different phases of the COVID-19 pandemic. For example, the correlation was significantly positive in phase 2 and late-phase 3 but was insignificant at other periods for both anxiety and overall mental disorders. Clear geospatial disparities in the prevalence of symptoms of mental disorders between the northern and southern United States were also found.

Our findings are consistent with those of previous research; Hussain [36] also identified the correlation between social media–based estimates and survey-reported prevalence by estimating public sentiment toward COVID-19 vaccines, using data from Facebook and Twitter platforms in United States and the United Kingdom and comparing the results with nationwide surveys [36]. Therefore, our findings could provide another piece of empirical evidence on the feasibility of using Twitter to estimate the prevalence of symptoms of mental disorders.

The correlations between Twitter-based and CDC-reported prevalence varied by different phases during the COVID-19 pandemic. The possible explanation is that Twitter-based prevalence is more time sensitive than the CDC-reported prevalence. The emotions potentially reflected through posting tweets are sensitive to daily events (eg, lifestyle change and staying at home) and social events (eg, citywide lockdown) [36,37]. However, the CDC-reported prevalence may lag behind the Twitter-based prevalence, which results in temporal trends in the correlation between them. Another explanation is that there may be a time lag between social events and some emotional responses. For example, Zhang et al [37] found that Twitter users with depression would respond to the pandemic and post tweets later than those without depression. This may indicate the heterogeneity in the emotional responses regarding posting tweets, which could affect the accuracy of Twitter-estimated symptoms of mental disorders at different time points and induce its temporal correlation with CDC-reported prevalence. Future studies are needed to consider the time lag and the heterogeneity in emotional responses when using tweets to estimate the prevalence of symptoms of mental disorders.

Maps with random effects showed clear spatiotemporal disparities in the prevalence of symptoms of mental disorders in states located in the north-central and northeast United States, which had relatively lower prevalence of mental disorder symptoms than those from other parts of the United States. The disparities may be related to variations in the COVID-19 pandemic in different parts of the United States. On the one hand, different locations have a varying burden of COVID-19. For example, Miller et al [38] mapped the disease and hospitalization burden and found that areas located in the western United States had a high concentration of the cumulative number of severe COVID-19 cases. The overwhelming public health or health care system may fail to control disease transmission, which may increase panic in the public. On the other hand, the geospatial disparities in socioeconomic status (SES) may also contribute to the pattern found in our study. People living in low-SES regions may be at high risk of financial difficulty during the COVID-19 pandemic owing to stay-at-home orders, citywide lockdowns, and social distancing measures. Low SES has been proved to be associated with a greater risk of psychopathology after the COVID-19 pandemic [39]. However, previous studies did not show a consistent spatiotemporal pattern. For example, socioeconomic attributes (eg, education level, income, and occupation) have been confirmed to have a significant association with sentiment among Twitter users, but the number of COVID-19 cases in a region did not show a significant sentiment association [40]; however, Whitney and Peterson [41] found a similar pattern among US youth to that reported in this study in which areas in the southern United States had a worse mental health status than those in the northern United States.

### Implementation

Our model could be used as a quick instrument for estimating symptoms of mental health disorders. In the future, once an estimate of the prevalence of the symptoms of a certain mental health disorder is obtained from Twitter (via keywords or any other advanced estimation models), the Twitter-based estimate and corresponding time can be factored into our model, and an estimate of surveillance-based prevalence can be obtained.

### Limitations

There are some limitations in our study, which need to be acknowledged. First, although we retrieved representative keywords from previous research, there were still other keywords that were not considered in our study, which may result in the underestimated prevalence of symptoms of mental disorders. Second, since tweets with our predefined keywords may also refer to unrelated topics [42], the keywords used in this study may misclassify Twitter users into different groups regarding the presence of symptoms of mental disorders, which may affect the accuracy of estimation and impair the validity of our findings. Future studies are needed to improve our strategy of refining tweets to obtain accurate estimations. Other advanced analytic approaches (eg, machine learning approaches) could be used in addition to more comprehensive keywords. Third, as an ecological study, the individual sociodemographic characteristics of Twitter users were not considered when estimating the prevalence of symptoms of mental disorders. Sociodemographic characteristics have a close relationship with the outcomes of mental disorders. Thus, incorporating confounders (eg, characteristics) into model training could improve the accuracy of model estimation [43-45]. Besides, other ecological confounders such as political proceedings and socioeconomic downfall are also not included in our study, and more efforts should be made to incorporate those confounders in future studies. Finally, the sociodemographics in Twitter data have a bias [30]. For example, compared to HPS participants aged 18 years and older, as most of the Twitter users tend to be young (18-29 years old) and in considerable SES [46], the mental health problems among them may be different from those of individuals who did not use Twitter. For instance, young high-SES adults may have better access to mental health resources or seek help proactively. Therefore, these populations may have a lower risk of experiencing symptoms of mental disorders than older lower-SES adults [47].

### Conclusions

Positive correlations were found between CDC-reported and Twitter-based symptom prevalence, and temporal trends of these correlations were identified. Spatiotemporal disparities were also observed between the northern and southern United States. Findings from this study could inform future research to improve the accuracy of estimating the prevalence of symptoms of mental disorders using social media platforms. Public health practitioners and policy makers could use Twitter-based prevalence to inform immediate prevention measures and mental health services to cope with mental disorders during the COVID-19 pandemic and future public health emergencies.

## Appendix

Multimedia Appendix 1 Mixed Models Coefficients.

Multimedia Appendix 2 Marginal Coefficient of Twitter-based Prevalence for Anxiety Symptoms.

Multimedia Appendix 3 Marginal Coefficient of Twitter-based Prevalence for Depressive Symptoms.

Multimedia Appendix 4 Marginal Coefficient of Twitter-based Prevalence for Overall Mental Disorder Symptoms.

## Abbreviations

CDC: Centers for Disease Control and Prevention
HPS: Household Pulse Survey
SES: socioeconomic status

## Data Availability

Twitter data were collected using Twitter’s public streaming application programming interface from the public domain in accordance with Twitter’s Developer Agreement. Following Twitter’s policy on “Redistribution of Twitter content” [48], individual tweets cannot be redistributed.
