# Supplementary material for: Using Twitter Data to Estimate the Prevalence of Symptoms of Mental Disorders in the United States During the COVID-19 Pandemic: Ecological Cohort Study
Source: JMIR Form Res. 2022 Dec 20;6(12):e37582. doi: 10.2196/37582 (PMC9770024; doi:10.2196/37582)
Supplement: Multimedia Appendix 1 [file formative_v6i12e37582_app1.docx]

| Appendix 1. Mixed Models Coefficients | | | | | | | | | | | | | | |
| --- | --- | --- | --- | --- | --- | --- | --- | --- | --- | --- | --- | --- | --- | --- |
|  | Anxiety Symptoms | | | |  | Depressive Symptoms | | | |  | Overall Mental Disorder Symptoms | | | |
|  | Phases | | | |  | Phases | | | |  | Phases | | | |
|  | (1) | (2) | (3) | (3.1) |  | (1) | (2) | (3) | (3.1) |  | (1) | (2) | (3) | (3.1) |
| Time | -0.023 | 0.554 | -0.678^***^ | -0.139 |  | 0.446^***^ | 0.278 | -0.384^***^ | -0.341^.^ |  | 0.163 | 0.201 | -0.876^***^ | -0.251 |
|  | (0.250) | (0.362) | (0.092) | (0.277) |  | (0.133) | (0.247) | (0.083) | (0.188) |  | (0.267) | (0.399) | (0.104) | (0.305) |
| Twitter | -2.703^*^ | 9.349 | -10.750^***^ | -1.646 |  | 3.676 | 4.361 | -29.016^**^ | -62.497 |  | -1.420 | 1.954 | -14.051^***^ | -5.376 |
|  | (1.153) | (6.392) | (2.760) | (16.447) |  | (2.483) | (16.959) | (11.120) | (46.831) |  | (1.069) | (5.791) | (2.582) | (15.085) |
| Time*Twitter | 0.527^*^ | -0.229 | 0.327^***^ | 0.029 |  | 0.293 | -0.256 | 0.848^**^ | 1.123 |  | 0.284 | 0.086 | 0.420^***^ | 0.089 |
|  | (0.206) | (0.299) | (0.089) | (0.296) |  | (0.415) | (0.811) | (0.295) | (0.842) |  | (0.181) | (0.271) | (0.083) | (0.272) |
| Constant | 30.625^***^ | 14.349^.^ | 58.048^***^ | 33.087^*^ |  | 21.001^***^ | 18.558^***^ | 40.945^***^ | 39.829^***^ |  | 34.407^***^ | 26.394^**^ | 70.212^***^ | 44.066^**^ |
|  | (1.435) | (7.865) | (3.083) | (15.426) |  | (0.892) | (5.293) | (3.237) | (10.465) |  | (1.633) | (8.674) | (3.489) | (16.984) |
| Observations | 588 | 245 | 490 | 294 |  | 588 | 245 | 490 | 294 |  | 588 | 245 | 490 | 294 |
| Note: . *P*<.1; * *P* <.05; ** *P* <.01; *** *P* <.001 | | | | | | | | | | | | | | |
