# Supplementary figures and images for: Using Twitter Data to Estimate the Prevalence of Symptoms of Mental Disorders in the United States During the COVID-19 Pandemic: Ecological Cohort Study
Source: JMIR Form Res. 2022 Dec 20;6(12):e37582. doi: 10.2196/37582 (PMC9770024; doi:10.2196/37582)

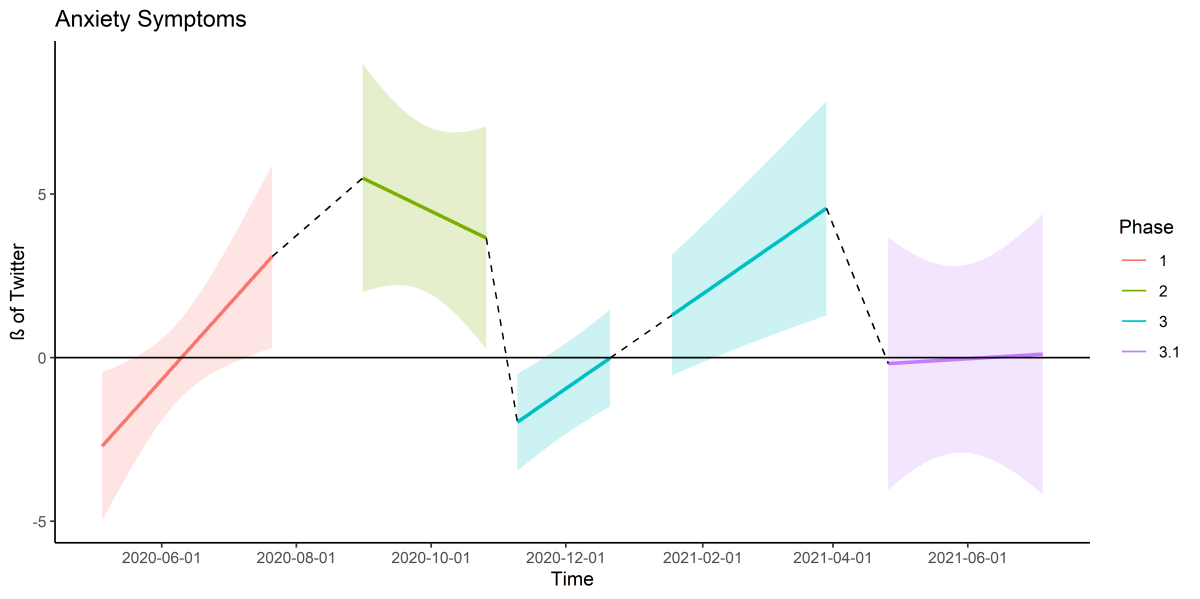

Supplement: Multimedia Appendix 2 [file formative_v6i12e37582_app2.png]

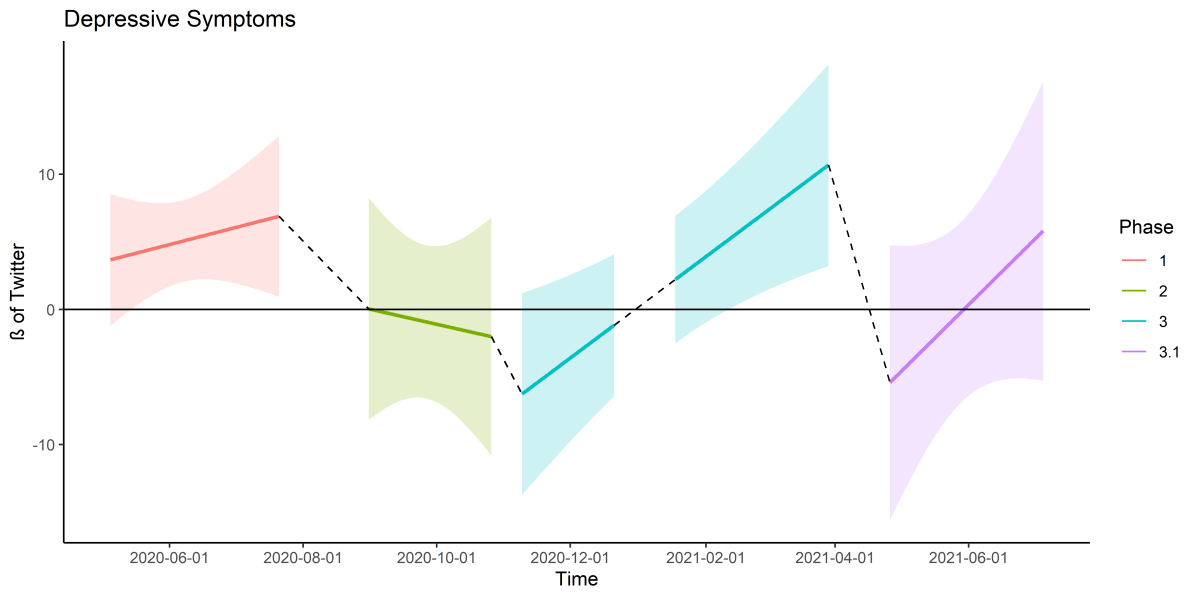

Supplement: Multimedia Appendix 3 [file formative_v6i12e37582_app3.png]

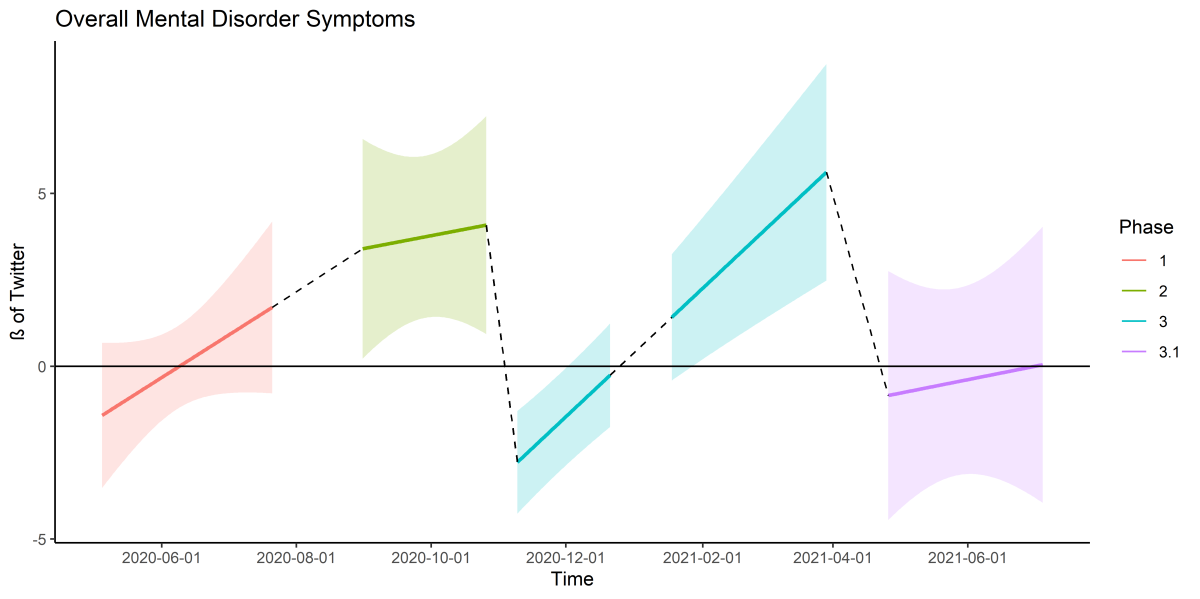

Supplement: Multimedia Appendix 4 [file formative_v6i12e37582_app4.png]
